# The Response of Thiols to Cadmium Stress in Spinach (*Spinacia Oleracea* L.)

**DOI:** 10.3390/toxics10080429

**Published:** 2022-07-28

**Authors:** Ya Gao, Haipu Li, Yang Song, Fenglin Zhang, Zhaoguang Yang

**Affiliations:** 1 Center for Environment and Water Resources, College of Chemistry and Chemical Engineering, Central South University, Changsha 410083, China; graceya333@outlook.com (Y.G.); trustwh2010@163.com (Y.S.); 15700711541@163.com (F.Z.); zgyang3@gmail.com (Z.Y.); 2 Key Laboratory of Hunan Province for Water Environment and Agriculture Product Safety, Changsha 410083, China

**Keywords:** glutathione, subtypes of phytochelatin, cadmium, spinach, response

## Abstract

The aim of this study is to examine the thiol species for the high cadmium (Cd) tolerance of spinach and provide information for the improvement of soil utilization. The spinach was cultured in aqueous solution with concentrations of Cd ranging from 1 to 9 mg/L. The time responses of glutathione (GSH) and phytochelatins (PCs, PC_2_-PC_4_) in the tissues of spinach were monitored via HPLC–MS/MS, and the concentrations of Cd in the roots, shoots and leaves were detected by ICP–OES. Data were analyzed via one-way ANOVA and Spearman correlation to assess the relationships among the types of thiols and the changes between types of thiols and Cd. As Cd stress increased, Cd concentrations in tissues also increased. The total thiol contents responded to Cd stresses with correlations r ranging from 0.394 (root), 0.520 (shoot) to 0.771 (leaf) (*p* < 0.01). GSH and PC_3_ were dominant on most of the days under Cd stress. The correlation *r* between improvements in GSH and increments of Cd concentration in roots was −0.808 (*p* < 0.01), and *r* between changes in PC_3_ and changes in Cd concentrations in leaves was −0.503 (*p* < 0.01). No correlation can be found between GSH and the subtypes of PCs in shoots, but strong positive correlations within the subtypes of PCs. Thiols can be produced in different tissues of spinach, while the shoots are only a transport tissue for GSH.

## 1. Introduction

It is of great concern that plants take in cadmium (Cd) from Cd-polluted agricultural soils that causes food safety issues [1], especially in Asia [2]. A nationwide survey of soil contamination was conducted between 2005 and 2013 covering more than 70% of China’s land area. Surface soil samples were collected from 8 × 8 km grids. Of these samples, 16.1% exceeded the Chinese environmental quality standard; for agricultural soils, the percentage of exceedance is even greater at 19.4%. Contamination by heavy metals and metalloids accounted for 82.4%. Among the heavy metals and metalloids, Cd ranks the first in the percentage of soil samples (7.0%), exceeding the Ministry of Environmental Protection limit [3]. Chinese soils are identified as being contaminated, with Cd contents of 0.3 and 0.6 mg/kg for soils with pH < 7.5 and >7.5, respectively [4]. According to a survey by the Ministry of the Environment of Japan, agricultural land designated as Cd-contaminated now exceeds 6000 ha throughout the country [5]. Cd occurs locally in high concentrations in the Northern Plains of the US [6] due to the background Cd soil concentrations, up to an average of 0.4 mg/kg [7] and a median of 0.3 mg/kg in the Canadian Great Plains [8]. Data showed higher concentrations of Cd in the agricultural soils of Western Europe compared with those in the new members of EU12 owing to phosphorus fertilizers [9]. Meanwhile, the agricultural areas in the EU are safe from Cd contamination; based on the analysis of the top soils from approximately 22,000 locations in EU; only 5.5% of the samples have concentrations above the threshold (0.3 mg/kg), and there was excess Cd in agricultural soils in isolated cases in France and Spain [10].

Cd is a nonessential toxic metal for plant physiological processes [11]. Once Cd enters plants, it will induce the overproduction of reactive oxygen species (ROS) and result in several adverse effects, e.g., decreasing the biomass, reducing the photosynthetic rate, and changing the uptake of nutrients [12]. There are two antioxidant defense strategies for scavenging excess ROS caused by Cd, accordingly contributing to maintaining redox homeostasis [13]. One is eliminating enzymatic antioxidants with the use of antioxidant/redox enzymes such as superoxide dismutase, catalase, peroxidase enzymes, monodehydroascorbate reductase, and dehydroascorbate reductase; the other is eliminating nonenzymatic antioxidants using antioxidant or redox metabolites, including ascorbate, glutathione (GSH, γ-Glu-Cys-Gly), and nicotinamide adenine dinucleotide phosphate to prevent the generation of and eliminate excess ROS [14,15,16]. Noticeably, in the metabolic process of GSH under Cd stress, phytochelatins (PCs, [(γ-Glu-Cys)n-Gly, where *n* = 2–11]) are synthesized to chelate Cd to less/nontoxic forms [17]. Subsequently, these Cd complexes can be transported into vacuoles for sequestration as an intracellular mechanism for Cd detoxification [18] that contributes to decreases in intracellular Cd concentration. GSH and PCs are thiol-peptide compounds with a sulfhydryl functional group (-SH) in their molecular structures [19].

Adamis et al. coded *GTT1* and *GTT2*, which can control the activities of GSH transferases. The results showed that the formation of the GSH–Cd complex is dependent on GSH transferases and simultaneously increases the tolerance to Cd [20]. Due to the similar structures of PCs and GSH, the process of PCs binding Cd to form PC–Cds also requires specific conditions. However, to date, direct evidence has been lacking of mediators of PCs to chelate Cd, which are the key to increasing Cd tolerance in plants. Fortunately, indirect evidence indicates that heavy metals can be chelated using different thiols with the catalysis of different mediators and under various conditions based on analyses of the differences in the PC–heavy metal complexes and the free PCs in plants. In sunflower, free GSH was the primary thiol rather than PCs, and of the PCs, PC_3_ and PC_4_ were more abundant than PC_2_ [21].

In an investigation of arsenic (As) stress, PC_2_–As was the dominant complex after 1 h exposure, but after 3 h, the predominant complex was PC_3_–As [22]. There was high free PC_4_ content under As stress but low PC_4_-As, reflecting that the utilization of PC_4_ was restricted by some mediators or that PC_4_ only can chelate Cd under specific conditions. In a marine microalga after 6 h exposure to Cd, the content of PC_2_ was equal to PC_4_, but both of them were less than the content of PC_3_. However, only PC_4_–Cd and PC_3_–Cd chelated Cd, and most PC_2_ in the cellular extract was collected in the metal-free form [23]; the finding indicated that the ligand of PC_2_–Cd needed to be mediated, but this mediation to bind PC_2_ and Cd was restricted in the marine microalga. When *Amaranthus hypochondriacus* was exposed to Cd stress, PC_2_–Cd and PC_4_–Cd were predicted to be the primary ligands [24], PC_2_ and PC_3_ were dominant in metal-free form in *Amaranthus hypochondriacus* [25], indicating that PC_3_ was partially utilized to chelate Cd for detoxication; however, PC_4_ was highly utilized for chelating Cd. Above all, only focusing on the contents of thiols and thiol–Cd complexes does not clearly reveal the mechanism of Cd tolerance in plants. To depict the restriction of binding thiols and Cd, the relationships between the improvement of different thiols and increments of Cd need to be observed. Meanwhile, because of the conversions among thiols, whether plants tend to produce more efficient thiols for chelating Cd is still unclear. Thus, combining this study with those studies on the responses of enzymatic antioxidants to Cd [26,27,28] can elucidate the reasons for Cd tolerance in spinach.

Previous studies showed that spinach is a Cd-tolerant crop and can be used for phytoremediation purposes [29]. Furthermore, the amounts of Cd in the leaves of spinach can be up to 367.7 mg/kg [30]. Spinach is one of the most highly consumed vegetables in the world. Food safety can be improved by decreasing the the amounts of Cd in spinach through restricting the reason for high Cd tolerance in spinach. Herein, by observing the time course of thiols (unbound GSH and subtypes of PC_2–4_) in various tissues upon Cd treatments ranging from 1 to 9 mg/L, the responses of total thiol and of different thiols to Cd concentrations were characterized. Reasons for the high Cd tolerance of spinach were found by analyzing the relationships between improvements in the thiols and increments of Cd concentrations. Additionally, the dominant thiol and the inner conversions among thiols were revealed.

## 2. Materials and Methods

### 2.1. Growth Condition and Treatment

After sprouting in the dark for 10 days, the seedlings of spinach were placed in a hydroponic incubator at 20 °C with a diurnal cycle of 16 h: 8 h, light: dark. The growth medium was a 70% Hoagland solution (full-strength composition 2.5 mM KNO_3_, 2.5 mM Ca(NO_3_)_2_·4H_2_O, 1 mM MgSO_4_·7H_2_O, 0.5 mM KH_2_PO_4_, 0.045 mM H_3_BO_3_, 0.01 mM MnCl_2_·4H_2_O, 0.8 μM ZnSO_4_·7H_2_O, 0.3 μM CuSO_4_·5H_2_O, 0.4 μM Na_2_MoO_4_·2H_2_O) and 0.015 mM Fe (as EDTA complex) at pH 6.3, and replaced every 3 days. Then, the 28-day-old seedlings were used for the Cd-exposed experiment with a range from 1 mg/L to 9 mg/L. During the trial, the nutrient solution was replaced every 3 days for constant concentration. All of the chemicals and reagents used in this study were purchased from Aladdin Reagent Co., Ltd. (Shanghai, China). Ultrapure deionized water (18.2 MΩ cm, Direct-Q3, Millipore SAS, Molsheim, France) was used in the experiments.

### 2.2. Sample Collection

The samples were collected on day 1, day 3, day 5, day 7, day 9 and day 14. The procedure was as follows: first, the roots were soaked in desorption fluid, and the plants were rinsed off Cd^2+^ with ultrapure deionized water. Then, parts of basal roots, shoots and basal leaves were frozen in liquid nitrogen for the measurement of different thiols. Finally, the remaining basal roots, shoots and basal leaves were freeze-dried immediately and grounded into powders for Cd concentration analysis.

### 2.3. Measurement of Contents of Thiols

The extraction of thiols followed the method of Yu [31]. The samples (200 mg) were mixed with 2 mL 100 mM dithiothreitol (DTT) (4 °C) in a 5 mL polypropylene centrifuge tube using a tissue homogenizer (T10 basic, IKA, Staufen, Germany). After vortexing for 1 min and sonicating (KQ-800TDE, Kunshan, China) for 5 min, the supernatant was sedimented at 4 °C for 10 min by centrifugation at 7000*× g* (Biofuge Primo R, Heraeus, Germany). Afterward, 0.8 mL supernatant was mixed with 0.1 mL acetonitrile and 0.1 mL formic acid and passed through a preconditioned polymer anion exchange resin (PAX) cartridge (1 mL, 30 mg). Finally, the extracts were filtered through 0.22 μm organic phase filters and analyzed immediately. 

The different thiols were separated and analyzed using Agilent 1260 series high-performance liquid chromatography (HPLC) system (Agilent, Santa Clara, CA, USA) coupled to a tandem G6460C electrospray triple–quadrupole mass spectrometry system (Agilent Technologies, GA, Santa Clara, CA, USA). Eclipse XDB-C18 column (2.1 × 150 mm, 3.5 μm) was used as an analytical column with connection to a guard column (5 × 4.6 mm, 2.7 μm, Poroshell 120 EC-C18, Agilent Technologies, USA). The gradient elution of HPLC conditions was optimized as follows: 0–8 min, linear gradient 10–50% B; 8.1–12 min, linear gradient 50–90% B; 12.1–16 min, isocratic 90% B; and the final gradient re-equilibrated the column to starting conditions from 16.1 to 20 min, where mobile phase A is 0.1% formic acid and solvent B is acetonitrile. The standards of the different thiols were obtained from Anaspec, San Jose, CA, USA. The total thiol was the sum of GSH and PC_2–4_. Quality assurance was performed by analyzing the 1 mg/L thiols. The contents of thiols in tissues are expressed as μg/kg fresh weight (FW).

### 2.4. Cd Concentration Analysis

Samples of 0.05 g were digested with 5 mL HNO_3_ at 120 °C for 2 h on a hot plate. After cooling, the samples were diluted to 15 mL with deionized water and analyzed using inductively coupled plasma–optical emission spectrometry (ICP–OES) (Optima 8000, PerkinElmer, Waltham, MA, USA). Quality assurance was performed by analyzing the SRM GBW10049 green onion (Institute of Geophysical and Geochemical Exploration, Chinese Academy of Geological Sciences, Langfang, China). The Cd concentrations in tissues are expressed as μg/kg dry weight (DW). 

### 2.5. Statistical Analysis

One-way ANOVA followed by the Tukey test or Dunnett’s T3 post hoc test (if variances were not homogenous) was conducted to estimate the statistical differences in the thiols between the control group and the Cd-exposed groups as well as the statistical differences in the Cd concentrations in the same tissue at different time points under the same Cd stress. The Spearman correlation analysis was performed to assess the response of total thiol to Cd concentrations in tissues, the improvements of thiols to increments of Cd concentrations in tissues, and the inner relationships of the thiols using SPSS software (IBM, Armonk, NY, USA).

## 3. Results

### 3.1. Contents of Thiols in Different Tissues

**Figure 1:**
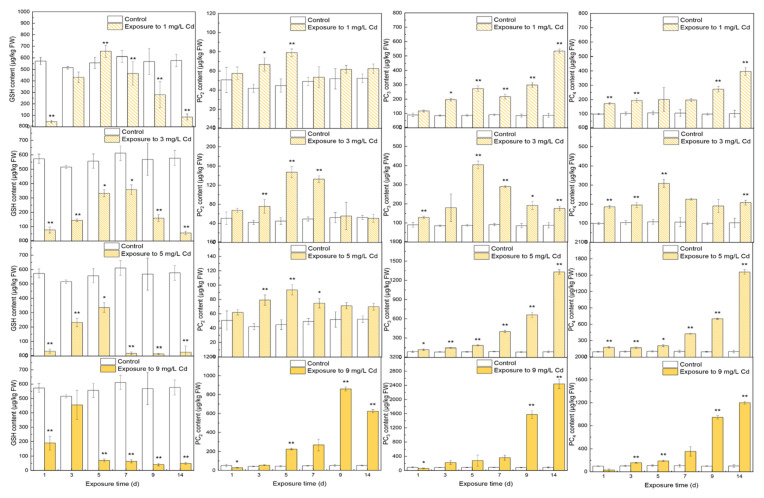
The contents of thiols in roots under different levels of Cd stress. All values represent the means ± SD of three replicates. Stars indicate significant differences from the control (**: *p* < 0.01, *: *p* < 0.05).

**Figure 2:**
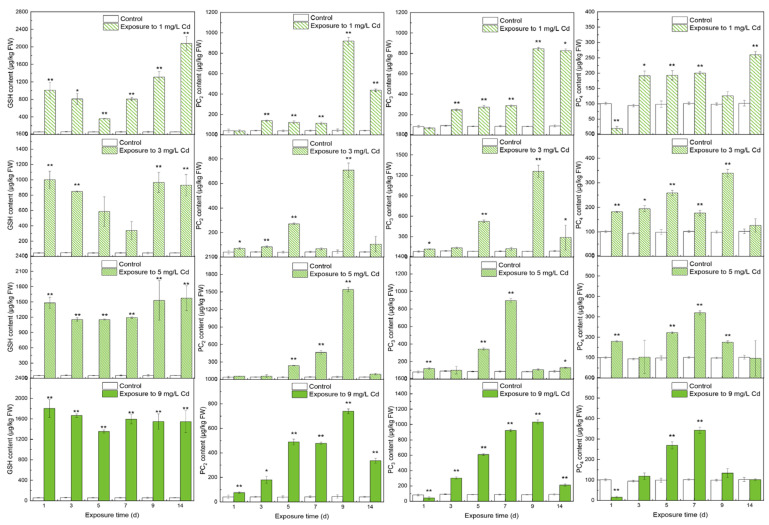
The contents of thiols in leaves under different levels of Cd stress. All values represent the means ± SD of three replicates. Stars indicate significant differences to the control (**: *p* < 0.01, *: *p* < 0.05).

**Figure 3:**
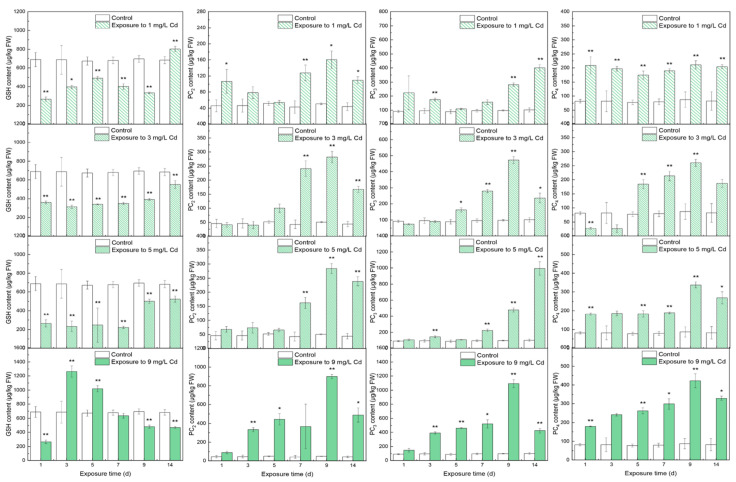
The contents of thiols in shoots under different levels of Cd stress. All values represent the means ± SD of three replicates. Stars indicate significant differences to the control (**: *p* < 0.01, *: *p* < 0.05).

The contents of thiols in different tissues under various Cd stresses are shown in Figure 1, Figure 2 and Figure 3. In roots, GSH increased and then decreased to stable contents with a significant difference from the control group (567.11 ± 28.40 μg/kg FW, *p* < 0.01). Noticeably, GSH contents were all less than those in the control group except on day 5 under 1 mg/L Cd stress. The free PC_2_ increased to peaks that showed significant differences from the control (*p* < 0.01) in all Cd-exposed groups. With 9 mg/L Cd treatment, PC_2_ peaked on day 9 at 860.16 ± 20.40 μg/kg FW. After reaching the peaks, PC_2_ decreased to a constant value thereafter. Viewing the time course of PC_3_, all samples peaked on day 14 with significant increases over the control (*p* < 0.01); as CD stress increased, the higher peaks ranged from 533.72 ± 14.05 μg/kg FW (1 mg/L Cd) to 2439.90 ± 132.94 μg/kg FW (9 mg/L Cd) except in the 3 mg/L Cd-treated group, with a peak of 404.46 ± 19.82 μg/kg FW on day 5. The responses of PC_4_ under various Cd stress levels were similar to those of PC_3_. 

In leaves, thiols were significantly higher under Cd stress than in the control group. GSH initially decreased and then returned to a relatively stable status with no significant differences in values between days 9 and 14 (*p* > 0.05). PC_2_ all peaked on day 9, with the highest value being 1542.62 ± 37.38 μg/kg FW under 5 mg/L Cd stress. All PC_3_ peaked on day 9 except under 5 mg/L Cd stress. The highest value was 1260.97 ± 89.26 μg/kg FW under 3 mg/L Cd stress. With the increasing of Cd stress, PC_4_ increased slightly and achieved lower peaks than the other thiols. The highest peak was at 342.75 ± 14.79 μg/kg FW under 9 mg/L Cd stress.

In shoots, the time courses of thiols were different from those in roots and leaves even though the shoots are the middle tissue of the plant. GSH contents were significantly lower than that in the control groups except on day 14 under 1 mg/L Cd stress and on day 9 under 9 mg/L Cd stress. PC_2_ under increasing Cd stress levels increased significantly (*p* < 0.01) and then peaked on day 9. Furthermore, with the increase of Cd stress, the peaks increased from 160.36 ± 22.20 μg/kg FW under 1 mg/L Cd stress to 898.54 ± 22.25 μg/kg FW under 9 mg/L Cd stress. With increasing Cd stress, PC_3_ significantly increased. The peaks of PC_3_ under 1 and 5 mg/L Cd stress were reached on day 9, and the others peaked at the end of the trial. The responses of PC_4_ were similar to PC_2_ under varying Cd treatment, but a fluctuation in PC_4_ was found under 1 mg/L stress. Noticeably, PC_3_ content in shoots was greater than that of the other PCs under the same Cd concentration.

### 3.2. Cd Concentrations in Different Tissues

**Figure 4:**
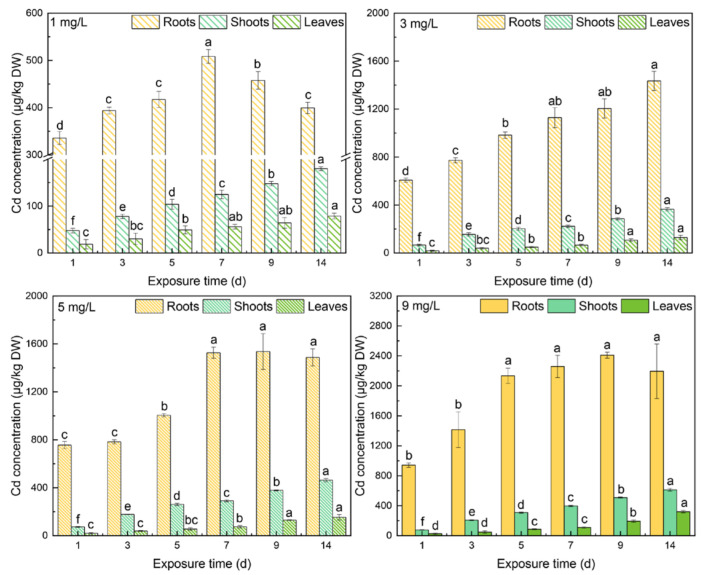
The Cd concentrations in different tissues under different levels of Cd stress. All values represent the means ± SD of three replicates. Significant differences (*p* < 0.05) at different time points in the same tissue are indicated with different letters.

The Cd concentrations in different tissues under varying levels of Cd stress are shown in Figure 4. With increasing Cd stress, Cd concentrations increased in tissues. As time went by, Cd concentrations in shoots and leaves kept increasing. The peaks of the shoots on day 14 from 1 mg/L to 9 mg/L Cd stress were 180 ± 4 μg/kg DW, 366 ± 14 μg/kg DW, 464 ± 14 μg/kg DW, and 613 ± 17 μg/kg DW. In the leaves, the peaks on day 14 were 79 ± 6 μg/kg DW, 130 ± 18 μg/kg DW, 153 ±23 μg/kg DW, and 321 ± 17 μg/kg DW. In the roots, meanwhile, Cd concentration increased with increasing Cd from 3 mg/L to 9 mg/L Cd treatment, but at 1 mg/L, Cd concentration peaked on day 7 and then decreased significantly. The peaks of roots under 1 mg/L to 9 mg/L Cd stress were 508 ± 15 μg/kg DW, 1435 ± 80 μg/kg DW, 1536 ± 150 μg/kg DW, and 2409 ± 41 μg/kg DW. Cd concentrations decreased from roots to leaves. Noticeably, the differences in Cd concentration in different tissues between under 3 and 5 mg/L Cd stress were much smaller than between under 1 and 3 mg/L Cd stress.

## 4. Discussion

### 4.1. The Responses of Thiols to Cd Concentrations

**Figure 5:**
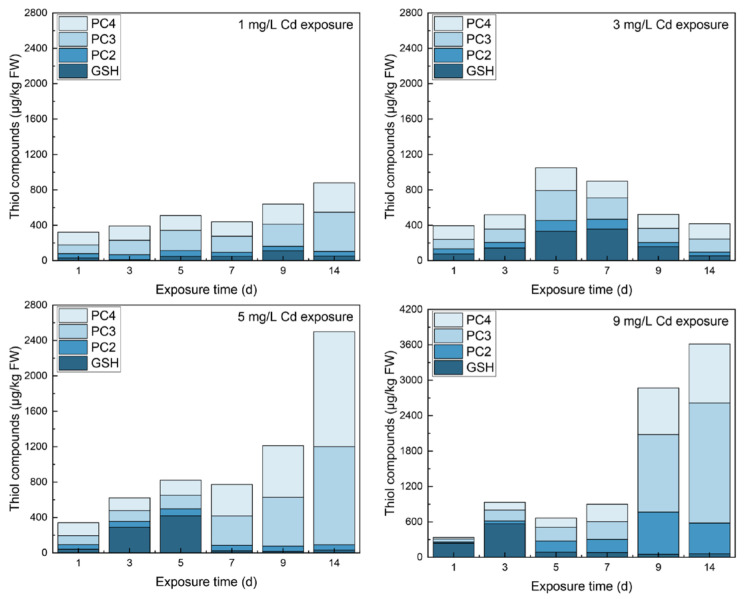
The total thiol in roots under different levels of Cd stress.

**Figure 6:**
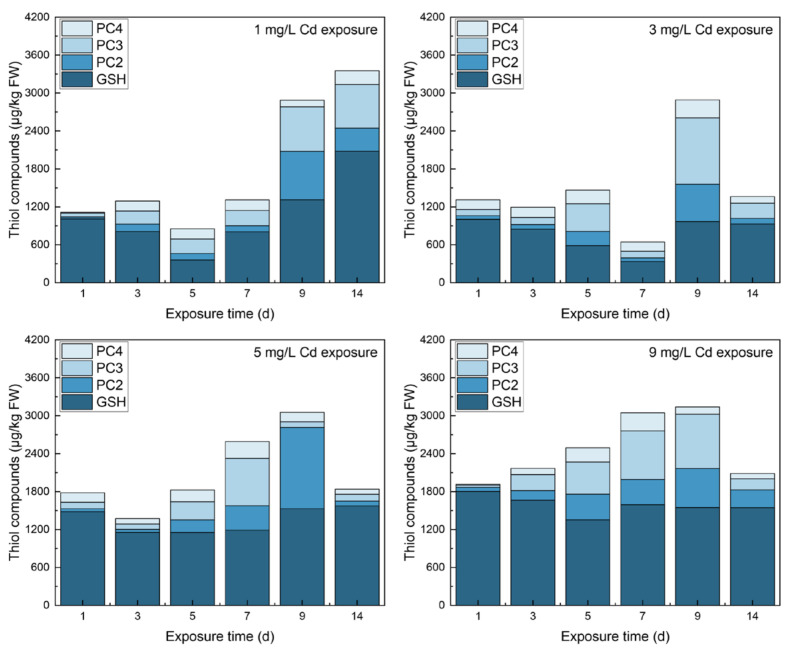
The total thiol in leaves under different levels of Cd stress.

When Cd is taken by plants, it is chelated by thiols to reduce ROS and toxicity [32] and maintain a sustainable redox state in plants [33]. The total thiol in different tissues of spinach under varying Cd stresses are shown in Figure 5, Figure 6 and Figure S1.

With increasing Cd concentrations in the tissues, total thiol increased in an antioxidative process. The total thiol responded to the Cd concentrations in each tissue with positive correlations at the significance of *p* < 0.01, and *r* from the roots to the leaves was 0.394, 0.520, and 0.771. The findings showed GSH and PC_2–4_ responding to Cd concentrations in spinach for the alleviation of ROS stress. Combining the *r* values and the phenomenon that the Cd concentrations in the roots dramatically declined beginning on day 7 under 1 mg/L Cd stress indicated that thiols help inhibit Cd transport from the tips of roots to basal roots, and thiols played crucial roles in antioxidative processes under low Cd stress. However, a decrease in Cd was only found under 1 mg/L Cd stress. It was inferred that there would be enzymatic antioxidant coupled with thiols [34] in the antioxidative processes caused by high Cd stress.

The content of total thiol is inaccurate for investigating the reason for Cd tolerance because there are many subtypes of thiols responding to Cd concentrations [35]. Due to the lack of information on the produced thiols, the activities of enzymes related to thiols, the conversions among thiols, and the responses of enzymatic antioxidants, there was little analysis of the time courses of thiols under different Cd concentrations. With GSH [36], enzyme activities all rose under increasing Cd stress, but GSH contents were lower on day 3 under 3 and 5 mg/L Cd stress. It meant that more Cd was chelated by GSH or more PC_2-4_ were synthesized from GSH under 3 and 5 mg/L Cd stress on day 3.

Because PCS activity was the highest under 5 mg/L Cd stress, GSH was synthesized to PC_2-4_ under 3 and 5 mg/L Cd stress on day 3. The higher content of PC_2-4_ in Figure 1 can also verify the phenomenon. Similarly, GSH content dropped on day 5 under 9 mg/L stress because the enzyme activities were low and stable since day 5. Because of PC_2_’s high peaks on day 9 and 14, it can be transferred from the other PC subtypes, i.e., PC_3_ and PC_4_, and it can be transported from other tissues. However, PCS activity was low in leaves [36]; transporting PC_2_ from leaves was almost impossible. Owing to the lack of enzyme activities related to GSH in the shoots, more PC_2_ may be produced and transported from shoots to roots. The change trends for PC_3_ and PC_4_ were similar, but the reason was unclear. It could be the transported PC_3-4_ from shoots or the conversions among the other of thiols. Though the time response of thiols cannot be analyzed, the reasons for high tolerance to Cd in spinach still can be determined. The thiol constituent contents in different tissues responding to varying Cd concentrations deserved more attention (See Figures S2–S4). The percentages of GSH occupied the principal position for only 3 days under 3–9 mg/L Cd stress in roots. PCs with more -SH for the chelation of Cd showed dominant responses to various Cd concentrations. Free PC_3_ and PC_4_ both occupied a larger portion compared with PC_2_ (Figure 5), and PC_3_ ranked the first place on most of the days during the trial. Similar results were also observed in *Arabidopsis*. Hendrix et al. studied the thiol levels of *Arabidopsis* under 5 μM Cd stress at a duration of 21 days [37]. The results showed that each subtype of PC_2_-PC_5_ contained more than GSH content, and the primary subtypes of PCs were PC_3_ and PC_4_ in roots.

In leaves, the storage of total thiol was less than that in roots, indicating that roots were more prepared to tolerate a serious imbalance of ROS between production and elimination [38]. However, the total thiol was higher in leaves than in roots when exposed to Cd stress. In an observation of Indian mustard under Cd stress, there were more of the different thiols in leaves than in roots [39]. This means that plants prefer to produce more thiols to detoxify Cd in leaves to survive because the major energy-carrying molecule ATP is produced in the chloroplasts and mitochondria of leaves [40]. The reason for the changing trend of GSH was discussed as resulting from the change trends of enzymes associated with GSH, although the reasons for the changing trends of PC_2–4_ were still unclear. The high content of PC_2_ can be the conversion from other thiols. The activities of enzymes in roots were low, and thus, little PC_2_ could be transport from roots; this caused the high PC_2_ content in the leaves. PC_3_ was increased on day 14 because its activity was higher than other enzymes. The reason for PC_4_ was unclear. The thiol constituents in leaves were different from in roots. The principal thiol was GSH in leaves compared with the subtypes of PCs responding to Cd concentrations in leaves (Figure 6). This phenomenon of thiol constituents also existed in Chinese cabbage with 12-day exposure to zinc, which shared the same uptake channel and valence electrons as Cd [41]. Thus, it is putatively determined that GSH is typically dominant under divalent heavy metal stress.

Above all, considering that thiols can transport bidirectionally in spinach, GSH and PC_3_ were the dominant thiols in response to Cd concentrations. With increasing Cd stress, higher concentrations of Cd were found in every tissue. Noticeably, when spinach was treated under 3 and 5 mg/L Cd stress, Cd concentrations in roots and leaves showed no significant differences on any day except on day 1. This verifies that some thiols can chelate Cd more efficiently so that the tolerance of spinach to Cd stress increases. Because the samples were collected from the basal parts of roots, Cd complexes can be transported into vacuoles for immobilization [42] in the remainder of the roots. However, the reason for the high tolerance to Cd in spinach remains unclear. Thus, the associations between changes in different types of thiols and increments of Cd concentrations in the same tissues were measured. It turned out that there was a very strong negative correlation between changes in GSH and increments of Cd concentration in roots, *r* = −0.808 (*p* < 0.01), and there was a moderate negative correlation between changes in PC_3_ and changes in Cd concentrations in leaves, *r* = −0.503 (*p* < 0.01), suggesting that GSH in roots and PC_3_ in leaves can increase the tolerance of Cd concentrations in spinach. Furthermore, there was no correlation between changes in Cd concentrations in roots and changes in different thiols in roots (*p* > 0.05); there were also no correlations between changes in Cd concentrations or in the different thiols in leaves. These findings indicated that the local thiols played insignificant roles under high Cd stress in spinach. Findings also suggest that thiols more effectively chelate Cd under Cd stress less than 5 mg/L; thiols should cooperate with enzymatic antioxidants for antioxidation under 9 mg/L Cd stress.

Though there was a dynamic antioxidative process when spinach was exposed to Cd stress, the contents of GSH and PC_3_ were dominant on most of the days, and GSH and PC_3_ effectively increased the tolerance to Cd accumulation in spinach when exposed to Cd stress less than 5 mg/L. Thus, food safety can be protected by decreasing GSH and PC_3_.

### 4.2. The Inner Conversions of Thiols

To reveal the inner relationships of the subtypes of thiols, Spearman’s correlation coefficient *r* among different subtypes of thiols are shown in Table 1.

There were negative correlations between GSH and subtypes of PCs in roots at a significance level of 0.01, indicating little GSH content as the substrate for the synthesis of PCs [43], and the production of GSH was less than the consumption of GSH due to the scant -SH groups [44]. Furthermore, moderate positive correlations among different subtypes of PCs in roots verified that the synthesis of PCs needs GSH as material [45]. There were positive correlations among the thiols in leaves, indicating that the production and storage of GSH were sufficient compared with the GSH content in roots under Cd stress. However, a comparison between the storage of GSH in roots and that in leaves showed that more GSH was produced in leaves or more GSH was transported from roots and shoots to leaves in the antioxidation of plants; the enzyme activities involved in the GSH content in roots were more active than that in leaves [46], implying that more GSH should be transported to leaves instead of being synthesized in local tissue. The correlation coefficient between GSH content in roots and in shoots was *r* = −0.327 (*p* < 0.01), and in leaves, it was *r* = 0.678 (*p* < 0.01), verifying that GSH should be transported from roots to leaves. This phenomenon was also observed in rice, with a greater abundance of GSH in shoots than in roots [45] because more GSH was transported from roots to shoots in response to the minimal amount of GSH content for use as the substrates for the synthesis of PC–GSH and PC–Cd in shoots. In spinach, strong positive correlations were observed between the subtypes of PCs, showing that PCs can be synthesized in shoots. However, no correlation can be found between GSH and the subtypes of PCs in shoots, implying that the role of local GSH was translocation not the synthesis of PCs.

The responses of the different thiols to the Cd concentrations in different tissues of spinach are shown in Table 2. GSH in shoots did not correlate with Cd concentrations in shoots, indicating that free GSH in shoots was translocated from other parts of spinach. Overall, the primary function of shoots was GSH transport, and PCs were responsible for responses to Cd concentrations in antioxidative processes. The reason for the transport of GSH without the synthesis of PCs is unknown. It could be low enzyme activities in GSH synthesis and more PCS activity so that GSH can be synthesized to PCs by transporting GSH between leaves and roots; however, this needs verification.

Previous research showed that as Zn stress increased, Zn^2+^ transport velocity from roots to shoots increased in *Phaseolus vulgaris* (L.) [47], suggesting that Zn^2+^ is easily transported from roots to the upper parts of plants. Due to the chemical similarity, the free Cd ion and Cd–PCs can also easily flow from roots to leaves in spinach. There was a strong positive correlation between Cd concentration in roots and leaves, with *r* = 0.834 at the significance of *p* < 0.01.

## 5. Conclusions

With increasing Cd stress, Cd concentrations in tissues increase, and the response of total thiol content to Cd also increases. Thiols can prevent and eliminate ROS under low levels of Cd stress, while thiols and enzymes can respond to high levels of Cd stress during the antioxidative process. The dominant thiols in spinach are GSH and PC_3_, which are also the reason for the high tolerance to Cd in spinach. PCs in spinach can be synthesized under Cd concentrations, but GSH can only be synthesized in roots and leaves.

## Figures and Tables

**Table 1:** The relationships among the contents of the thiols in the different tissues.

Tissue	GSH-PC_2_	GSH-PC_3_	GSH-PC_4_	PC_2_-PC_3_	PC_2_-PC_4_	PC_3_-PC_4_
Roots	−0.374 **	−0.485 **	−0.601 **	0.699 **	0.691 **	0.933 **
Shoots	0.052	0.094	0.053	0.908 **	0.904 **	0.928 **
Leaves	0.607 **	0.439 **	0.230 **	0.842 **	0.626 **	0.763 **

*: *p* < 0.05; **: *p* < 0.01.

**Table 2:** The responses of different thiols to Cd concentrations in different tissues.

Tissue	GSH	PC_2_	PC_3_	PC_4_
Roots	−0.609 **	0.662 **	0.687 **	0.678 **
Shoots	−0.105	0.832 **	0.816 **	0.790 **
Leaves	0.642 **	0.786 **	0.672 **	0.424 **

*: *p* < 0.05; **: *p* < 0.01.

## Data Availability

Not applicable.

## Supplementary Materials

The following supporting information can be downloaded at: https://www.mdpi.com/article/10.3390/toxics10080429/s1, Figure S1: Total thiol in shoots with treatment of varying concentrations of Cd; Figure S2: The constitutes of thiol compounds in roots under varying Cd stresses; Figure S3: The constitutes of thiol compounds in shoots under varying Cd stresses; Figure S4: The constitutes of thiol compounds in leaves under varying Cd stresses.
