# Supplementary material for: The Response of Thiols to Cadmium Stress in Spinach (Spinacia Oleracea L.)
Source: Toxics. 2022 Jul 28;10(8):429. doi: 10.3390/toxics10080429 (PMC9415539; doi:10.3390/toxics10080429)
Supplement: Supplementary file 1 [file toxics-10-00429-s001.zip › toxics-1833140-supplementary.pdf]

Supplementary Information

The response of thiols to cadmium stress in spinach (*Spinacia oleracea L.*)

Ya Gao,[1,2] *Haipu Li,* [1,2,*] *Yang Song*, [1,2] *Fenglin Zhang,* [1,2] *and Zhaoguang Yang*[1,2]

[1]Center for Environment and Water Resources, College of Chemistry and Chemical Engineering, Central South University, Changsha 410083, China

[2]Key Laboratory of Hunan Province for Water Environment and Agriculture Product Safety, Changsha, China

*Corresponding authors. Tel: +86 731 88876961

*Email addresses: lihaipu@csu.edu.cn (Haipu Li)

Tables and figures captions:

Figure S1 Total thiol in shoots with treatment of varying concentrations of Cd.

Figure S2 The constitutes of thiol compounds in roots under varying Cd stresses.

Figure S3 The constitutes of thiol compounds in shoots under varying Cd stresses.

Figure S4 The constitutes of thiol compounds in leaves under varying Cd stresses.

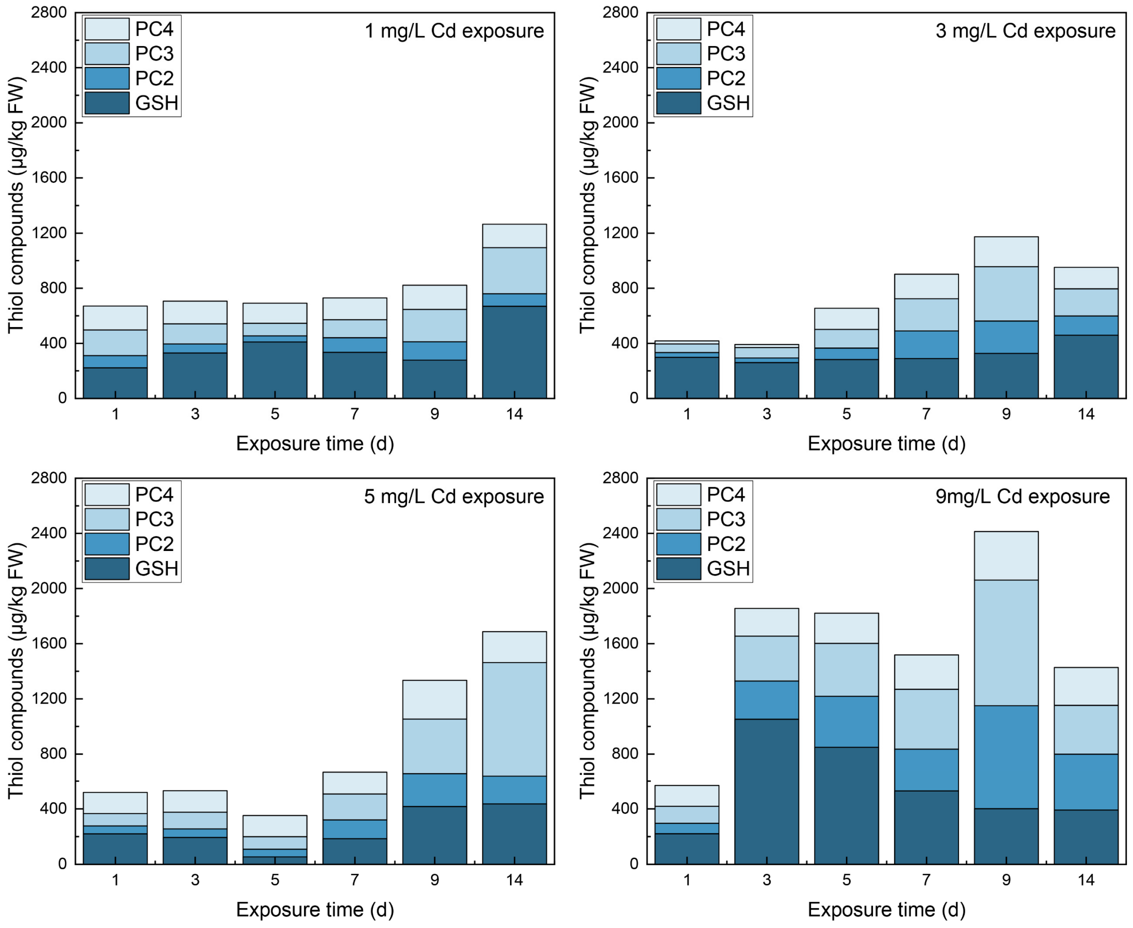

Figure S1 Total thiols in shoots with treatment of varying concentrations of Cd.

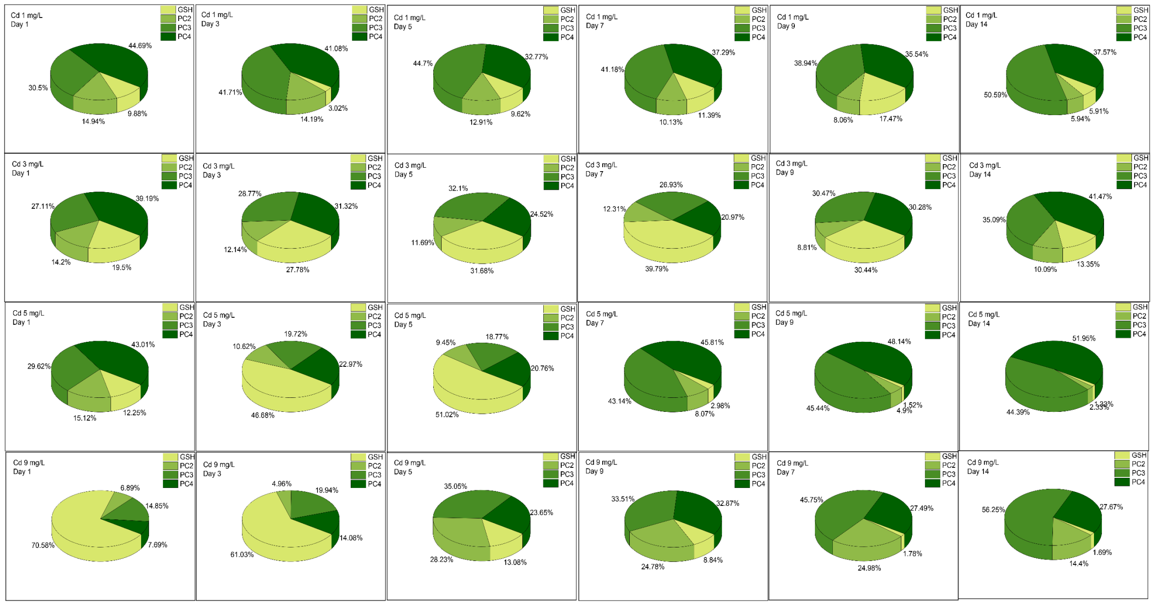

Figure S2 The constitutes of thiol compounds in roots under varying Cd stress.

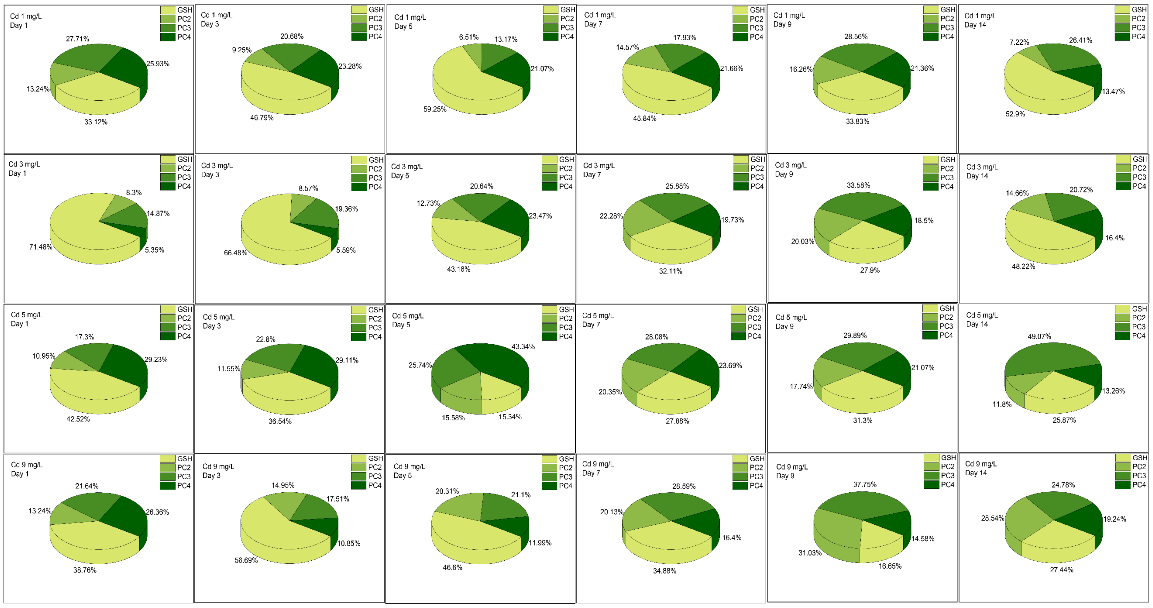

Figure S3 The constitutes of thiol compounds in shoots under varying Cd stress.

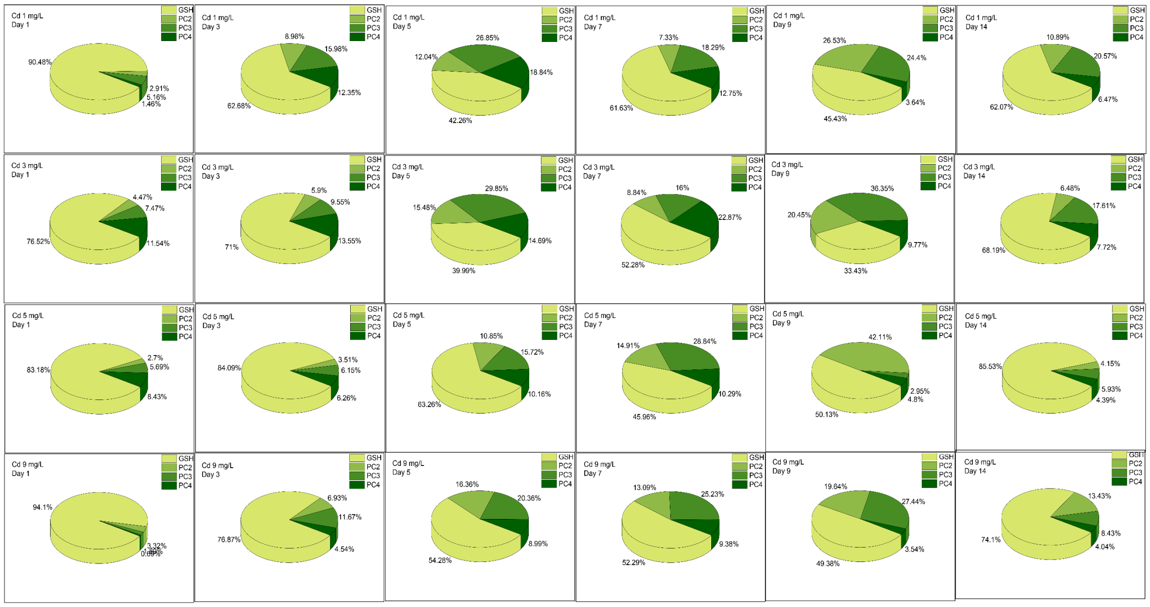

Figure S4 The constitutes of thiol compounds in leaves under varying Cd stress.
